# Using Mitogenomic and Nuclear Ribosomal Sequence Data to Investigate the Phylogeny of the *Xiphinema americanum* Species Complex

**DOI:** 10.1371/journal.pone.0090035

**Published:** 2014-02-27

**Authors:** Inga A. Zasada, Amy Peetz, Dana K. Howe, Larry J. Wilhelm, Daravuth Cheam, Dee R. Denver, Ashleigh B. Smythe

**Affiliations:** 1 USDA-ARS Horticultural Crops Research Laboratory, Corvallis, Oregon, United States of America; 2 Department of Zoology, Oregon State University, Corvallis, Oregon, United States of America; 3 Department of Biology, Hamilton College, Clinton, New York, United States of America; George Washington University, United States of America

## Abstract

Nematodes within the *Xiphinema americanum* species complex are economically important because they vector nepoviruses which cause considerable damage to a variety of agricultural crops. The taxonomy of *X. americanum* species complex is controversial, with the number of putative species being the subject of debate. Accurate phylogenetic knowledge of this group is highly desirable as it may ultimately reveal genetic differences between species. For this study, nematodes belonging to the *X. americanum* species complex, including potentially mixed species populations, were collected from 12 geographically disparate locations across the U.S. from different crops and in varying association with nepoviruses. At least four individuals from each population were analyzed. A portion of the 18S nuclear ribosomal DNA (rDNA) gene was sequenced for all individuals while the internal transcribed spacer region 1 (ITS1) of rDNA was cloned and 2 to 6 clones per individual were sequenced. Mitochondrial genomes for numerous individuals were sequenced in parallel using high-throughput DNA sequencing (HTS) technology. Phylogenetic analysis of the 18S rDNA revealed virtually identical sequences across all populations. Analysis of ITS1 rDNA sequences revealed several well-supported clades, with some degree of congruence with geographic location and viral transmission, but also numerous presumably paralogous sequences that failed to form clades with other sequences from the same population. Analysis of mitochondrial DNA (mtDNA) indicated the presence of three distinct monophyletic clades of *X. americanum* species complex nematodes. Two clades contained nematodes found in association with nepovirus and the third contained divergent mtDNA sequences from three nematode populations from the western U.S. where nepovirus was absent. The inherent heterogeneity in ITS1 rDNA sequence data and lack of informative sites in 18S rDNA analysis suggests that mtDNA may be more useful in sorting out the taxonomic confusion of the *X. americanum* species complex.

## Introduction

Nematode-vectored viruses occur worldwide and affect a range of host plants. Only two orders of nematodes, Dorylaimida and Triplonchida, contain members that vector viruses. Within the order Dorylaimida, there are three genera in the family Longidoridae that transmit viruses: *Xiphinema*, *Longidorus*, and *Paralongidorus*. In the genus *Xiphinema*, there is a taxonomically-unresolved group of economically important virus-vectoring nematodes known as the *Xiphinema americanum* species complex. This species complex is comprised of nematodes ranging in adult length from 1.3 to 3.0 mm that are soil inhabiting migratory ectoparasites, spending their entire lives outside the root. Upon feeding on roots, *X. americanum* species complex nematodes can vector four members of the genus *Nepovirus* (NEPO; *Tomato ringspot virus*, *Tobacco ringspot virus*, *Peach rosette mosaic virus*, and *Cherry rasp leaf virus*); nepoviruses are spherical, single-stranded RNA of positive-sense [1], [2].

Although *X. americanum* causes little damage when nepoviruses are not present, growers may choose to apply chemicals when *X. americanum* is detected in preplant soil samples because of the risk posed by the nematode-virus complex. Nepovirus infection is insidious in nature. As such, perennial crops are often symptomless for years post infection before replacement is necessary, which can be economically crippling to the grower. *Tomato ringspot virus* is one of the most economically-important viral diseases of red raspberry in North America and also affects blueberry, apple, and grape, while *Cherry rasp leaf virus* is of importance in cherry [3], [4]. In the United States, members of the *X. americanum* species complex are widespread [5], but this distribution does not always coincide with the presence of nepoviruses. It is also known that not all members of the *X. americanum* species complex can serve as vectors of nepoviruses [6]. The apparent lack of specificity in the transmission of North American nepoviruses by members of the *X. americanum* species complex contrasts with the specificity between European nepoviruses and their vector nematode species [7], [8]. Compared to other virus-vectoring members of the order Dorylaimida, *Longidorus* and *Paralongidorus*, *Xiphinema* have proven to be much more difficult to define at the species level. There are nine species of *Xiphinema* reported to transmit viruses; of these nine species, six are part of the *X. americanum* species complex (*X. americanum*, *X. bricolensis*, *X. californicum*, *X. intermedium*, *X. rivesi*, and *X. tarjanense*) [1], [9]. Since the original description of *X. americanum* sensu stricto [10], there has been a proliferation of species, up to 50 [11]–[13], within this group which is collectively referred to as *X. americanum* sensu lato or the *X. americanum* species complex.

Because of the minimal inter-and intra-specific variation in both morphological and morphometric characters in members of the *X. americanum* species complex, taxonomic clarification of this group may not be realized until it is well characterized at the molecular level [14]. Multiple regions of the ribosomal DNA (rDNA) genes [28S, 18S, and 5.8S genes and internal transcribed spacers (ITS1 and ITS2)], as well as the cytochrome oxidase 1 (COI) region of the mitochondrial (mt)DNA have been investigated attempting to better understand the taxonomic relationships within the *X. americanum* species complex. Sequence data from the 28S D2/D3 expansion region of the rDNA revealed very little intra-specific variation between *X. americanum* species complex populations, including virus-transmitting *X. tarjanense*, from Florida [15]. However, more recently phylogenetic analysis of the D2/D3 expansion regions revealed two well supported clades of *X. americanum* species complex nematodes [16]. The 18S region of the rDNA did not differentiate the virus-vectoring *X. americanum*, *X. rivesi*, and *X. tarjanense* [17], or several other non-virus-vectoring *X. americanum* species complex members [18]. It appears that the ITS region of the rDNA may be more informative. Restriction length polymorphisms in a region spanning the rDNA 5.8S gene and ITS regions arranged 16 *X. americanum* species complex populations into five subgroups [19]. However, these subgroups did not group virus-transmitting populations together, with *X. bricolensis* and *X. rivesi* being found in different phylogenetically-supported subgroups. Sequence data from the ITS1 region also identified subgroups placing *X. americanum* species complex populations together but separate from *X. italiae* and *X. diversicaudatum*, both of which are also virus-transmitting nematodes [20]. Also of interest is the quickly evolving mtDNA. Examination of the mtDNA COI region revealed that a dichotomy existed between *X. americanum* species complex populations from North America and those from Asia, South America, and Oceania [17]. This finding was not supported in subsequent phylogenetic analyses of partial COI sequence [16]. Our study provides a molecular phylogenetic analysis of *X. americanum* species complex nematodes from the U.S., collected from agricultural sites where host plants were scored for nepovirus infection that integrates analytical results from slow-evolving 18S nuclear ribosomal DNA locus (18S), fast-evolving internal transcribed spacer region 1 (ITS1) of rDNA regions, and fast-evolving mitochondrial DNA (mtDNA) regions obtained using a multiplex Illumina DNA sequencing approach.

## Materials and Methods

### *Xiphinema americanum* Populations

Populations were collected from twelve geographically disparate locations across the United States (Table 1). Samples were collected from various locations by university extension personnel or researchers (T. Kirkpatrick, University of Arkansas; J. Halbrendt, Pennsylvania State University; R. Pokharel, Colorado State University; Mark Fuchs, Cornell University; M. Ellis, Ohio State University). Permission was obtained by each of these individuals to enter grower fields or research sites as needed. The presence of nepovirus at these locations was based upon ELISA detection, plant symptomology, and/or historical information about the location. Soil was collected from the rhizosphere of host plants and sent to USDA-ARS in Corvallis, OR for extraction. Nematodes were extracted using a combination of decanting and sieving and Baermann funnel methods [21]. Extracted nematodes were examined to confirm that morphological characteristics were consistent with members of the *X. americanum* species complex sharing the characteristics of an offset lip region and conoid tail [22]. Extracted nematodes were hand-picked, placed in 1 M NaCl and stored at −20°C until processed.

**Table 1:** *Xiphinema americanum* species complex populations included in this study.

*Designation*	*Location*	*Crop*	Viruspresent^a^
AR-1	Hope, Arkansas	Blackberry	Yes
CO-1	Palisade, Colorado	Cherry	Yes
CO-2	Mesa, Colorado	Cherry	Yes
NC-1	Fletcher, North Carolina	Raspberry	Yes
NY-1	Geneva, New York	Cherry	Yes
OH-1	Wooster, Ohio	Blueberry	Yes
OR-1	Rogue Valley, Oregon	Grapevine	No
OR-2	Veneta, Oregon	Grapevine	Yes
PA-1	Biglerville, Pennsylvania	Apple	Yes
WA-1	Zillah, Washington	Grapevine	No
WA-2	Patterson, Washington	Grapevine	No
WA-3	Woodland, Washington	Blueberry	Yes

a Presence of nepovirus was based upon ELISA detection, direct observation of symptoms, or historical data for the site.

### DNA Extraction

DNA was extracted from a crude lysate from at least four individuals from each population following a modified established protocol [23]. A single nematode was placed in 10 µl of worm lysis buffer (WLB) [10 mM Tris pH 8.2; 2.5 mM MgCl_2_; 50 mM KCl; 0.45% Tween 20; 0.05% gelatin; 60 µg/ml proteinase K] and sliced into three segments on a sterile depression slide. Nematode segments were then mixed gently with an additional 10 µl of WLB, transferred to a 0.2 ml tube and subsequently frozen at −80°C for at least 30 min. The sample was thawed and 1.2 µl of a 1∶10 dilution of proteinase K in molecular biological grade H_2_O was added. The samples were then placed in a Veriti® 96-well Thermal Cycler (Applied Biosystems, Carlsbad, CA) at 60°C for 60 min followed by a 15 min incubation at 95°C to denature the proteinase K. Samples were stored at −20°C until processed.

### PCR and Sequencing Analysis of 18S rDNA

18S rDNA gene sequences were analyzed using a polymerase chain reaction (PCR) approach whereby ∼1.6 kb was amplified as two overlapping PCR amplicons, each ∼850 bp in length. The PCR primers described by Oliveira et al. [18] were used for amplification. PCR primers were also used to directly sequence amplicons using conventional fluorescent capillary sequencing technology. Sequencing was done with BigDye® Terminator v. 3.1 Cycle Sequencing Kit (Applied Biosystems) using an ABI Prism®3730 Genetic Analyzer and ABI Prism®3730 Data Collection (version 3.0) and DNA Sequencing Analysis (version 5.2) Software (Applied Biosystems), at the Center for Genome Research and Biocomputing (CGRB; Oregon State University, Corvallis, OR). DNA sequences were edited, aligned, and a consensus sequence was compiled using Geneious version 5.8 created by Biomatters (http://www.geneious.com/).

### PCR, Cloning, Sequencing, and Analysis of ITS1

PCR products of the entire ITS1 region, as well as the 5′ region of the 5.8S gene, were amplified using primers rDNA2 (5′-TTGATTACGTCCCTGCCCTTT-3′) and rDNA5.8S (5′-ACGAGCCGAGTGATCCACCG-3′) [24]. PCR reactions contained 2 µl of lysate solution in final volume of 25 µl. The PCR mixture included 11 µl sterile, distilled, DNase free, molecular grade H_2_O, 10 µl HotMasterMix (2.5x) (5 PRIME, Gaithersburg, MD), and 0.5 µl of each 10 µM primer. Each PCR was carried out using a Veriti® 96-well Thermal Cycler. Thermal conditions for PCR amplification after an initial denaturation step (94°C for 2 min) were as follows: 29 cycles at 94°C for 45 sec, 53°C for 40 sec, 65°C for 1.5 min; final extension step at 65°C for 10 min. PCR products, 3 µl, were analyzed on 1% agarose gels with ethidium bromide (1 µg/ml) in 1×TBE.

The PCR product was gel isolated with a QIAquick Gel Extraction Kit (Qiagen, Valencia, CA) and then cloned into pCR™4-TOPO® TA cloning vector (Invitrogen, Grand Island, NY) according to the manufacturer’s protocol. Plasmids were purified using a Wizard® Plus Minipreps DNA Purification System (Promega, Fitchburg, WI) using the recommended protocol. Plasmids were analyzed for proper transformation by restriction digest using *Eco*R I (Invitrogen). DNA sequencing and analysis was performed using the methods previously described for 18S rDNA sequences.

Newly generated ITS sequences (204; Table S1) from 49 individual *X. americanum* and 4 sequences obtained from GenBank (2 of *X. americanum*, 1 *X. citricolum* and 1 *X. peruvianum*) were aligned in ClustalW [25] using default parameters. *Xiphinema peruvianum* was designated as an outgroup. The phylogenetic analysis of 966 sites was conducted in Mega5 [26] using a maximum likelihood (ML) bootstrap with 1,000 replications and using the Kimura 2-parameter (G+I) model [27]. Nucleotide diversity (π; [28]) and its standard deviation were calculated using DnaSP [29] for aligned sequences within the 12 populations and 49 individual nematodes. As DnaSP does not allow IUPAC ambiguity codes in input data, ambiguities were designated as “N” and not used in calculations of π.

### mtDNA Amplification, HTS Experiments and Analysis

A long PCR approach was developed to amplify the entire mitochondrial genome of *X. americanum* in two amplicons. Details about this PCR approach for mtDNA were previously described in Howe and Denver [30]. The Expand Long Range PCR kit (Roche, Basel, Switzerland) with primers in the large and small rRNA genes and *trnR*, designed from the published *X. americanum* mitochondrial genome sequence [31] was used. The primers for long PCR (Xa_lsuA_F: CAACATCGAGGTCAACTATTC; Xa_ssu_R: ATCTGTTATGGACCGAAGAAG; Xa_Arg_F: TTAGTGGGTTACTACGCTTGG; Xa_lsuA_R: AGAATAGTTGACCTCGATGTT) produced two amplicons (approximately 7,800 kb and 7,100 kb) which together covered the entire mitochondrial genome.

The long PCR amplicons were used as templates for Illumina HTS. Approximately equal concentrations of the two amplicons were mixed for each nematode sample (150–300 ng/µL). Libraries were prepared following Illumina’s genomic DNA protocol, with the exception that custom barcode sequence-containing adapters were ligated to each strain individually [32]. We used 48 distinct barcoded adapters, one for each of the 48 nematodes targeted (4 individual nematodes from each of 12 populations – see Results) with long PCR. Each sample was quantified at the end of the library prep, and roughly equal amounts (∼500 ng/µL) of each library were pooled into one sample for sequencing on an Illumina GAII machine at the CGRB. The pooled libraries were loaded into one flowcell lane for a 160 bp single-end run.

The Illumina reads were bioinformatically parsed according to the sample-specific barcodes for each strain. A combination of both *de novo* assemblies and reference-guided assemblies to obtain mtDNA contigs used for downstream analyses were performed. Sample-specific reads were subject to *de novo* assembly using SCRAPE, a Perl-based assembly program compiler developed in the Denver lab by LJW. SCRAPE provides a two-stage assembly pipeline. In the first stage, *de novo* assembly software applications developed for short-read Illumina data are applied in multiple iterations to generate initial mtDNA contigs. Applications used in this first stage include Velvet [33], ABySS [34], Edena [35], and SSAKE [36]. Contigs resulting from the first-stage assembly process are then used as input for the second stage of the assembly process which utilizes CAP3 [37], which is optimized for assembling larger stretches of DNA sequence. SCRAPE.pl is freely available for download on the Denver lab website: denverlab.cgrb.oregonstate.edu.

Using SCRAPE, three *X. americanum* samples were each assembled into a single contig that constituted the complete mtDNA genome, as compared to the published *X. americanum* mtDNA sequence [31]. These sequences, along with the GenBank reference, were used for reference-guided assembly in CLC Genomics Workbench (CLC Bio, Aurhus, Denmark). After extensive *de novo* (both in SCRAPE and CLC) and reference-guided (in CLC) assembly runs, we achieved strong coverage of mtDNA sequence for many samples, though weak to no coverage for others (Table S2). Large single contigs containing many mtDNA coding gene sequences were obtained for 32 out of 48 samples (Table S2). These contigs were used for subsequent phylogenetic analyses (Table S1).

**Figure 1:**
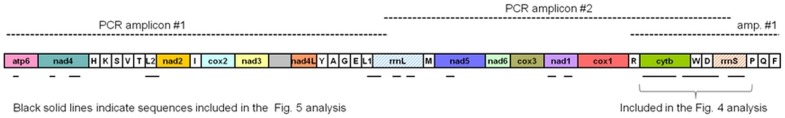
Schematic of loci of mitochondrial DNA analyzed. Rectangles with solid colors show mtDNA protein-coding genes, dashed rectangles show ribosomal RNA genes, and white rectangles show tRNA genes (single-letter abbreviations for associated amino acids). The dashed lines on top indicate the regions amplified in the two long PCRs used to generate templates for Illumina sequencing. Information about regions used for phylogenetic analyses is provided at the bottom.

Phylogenetic analyses for mtDNA were performed using MEGA5. We performed phylogenetic analyses on two different aligned mtDNA data sets, one optimized to include a maximum number of nematode samples (n = 32) at the cost of bp sites analyzed (∼1,130 bp), and a second that included more bp sites (∼2,565 bp) but fewer nematode samples (n = 13, plus the GenBank sequence accession NC_005928; [31]), though representing all the major lineages identified in the earlier analysis. Prior to phylogenetic analyses, the optimal model was determined for molecular evolution to be applied in each case using MEGA5. For the first analysis of 32 samples, the largest mtDNA contigs were aligned using MUSCLE in MEGA5 and regions with sequence coverage were concatenated, providing ∼1,130 bp between the *trnR* and rrnS genes (Figure 1). A maximum likelihood analysis using the Tamura 3-parameter+G model (with 1,000 bootstraps) was performed. For the subsequent analysis of 13 samples a similar maximum likelihood analysis, though using the Hasegawa-Kishina-Yano+I model (with 1,000 bootstraps), was performed.

## Results

### Analysis of 18S rDNA

We first analyzed patterns of molecular variation in ∼1.6 kb of 18S rDNA gene sequence. These sequences were amplified as two overlapping PCR amplicons (each ∼850 bp), using previously described approaches [18], [38], from four different nematode representatives for each of the twelve populations examined in this study. These 48 nematodes were the same samples that were analyzed using the mitogenomic approach, described later. For the vast majority of samples (44/48), the 18S rDNA sequences analyzed were identical to one-another and to the *X. americanum* GenBank entry (accession AY283170; [38]). The four representative nematodes from the PA-1 population shared a single polymorphic site (A present instead of G in the other 44 samples) that distinguished them from the other samples analyzed.

### Phylogenetic Analyses of Internal Transcribed Spacer Region rDNA (ITS)

**Figure 2:**
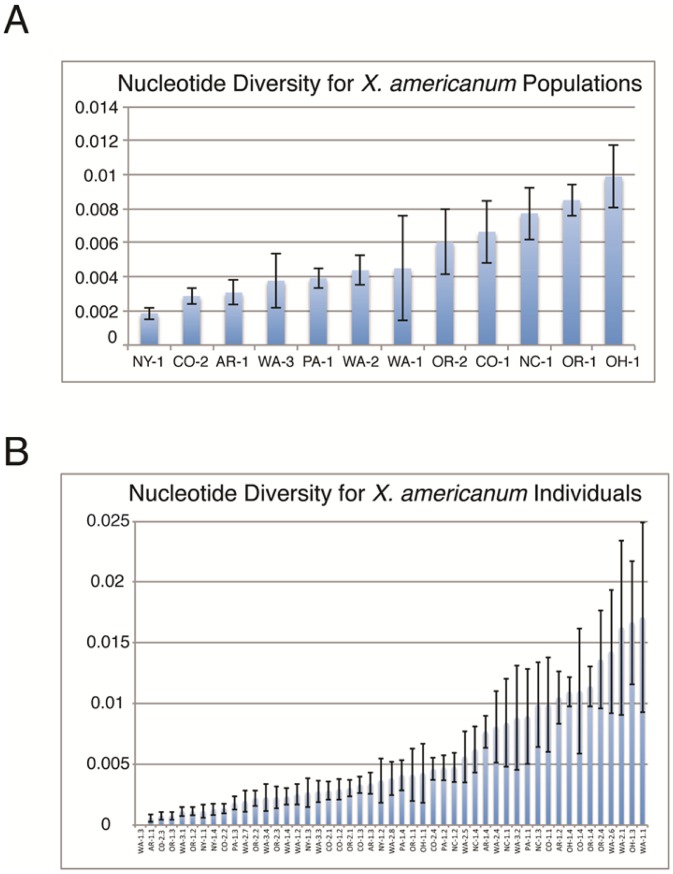
Nucleotide diversity (π) of ITS sequences from 12 populations of *X. americanum* (A) and 49 individual *Xiphinema americanum* (B) from North America. Error bars represent the standard deviation of π. Individual nematodes are indicated with a number following the population designation (e.g. WA-1.3 represents individual 3 from population WA-1). Note that the y-axes differ in scale from A (populations) to B (individuals).

We amplified the ITS1 locus from 49 *X. americanum* species complex nematodes from 12 populations, cloned the PCR products and sequenced between 2 and 6 clones per nematode. Maximum likelihood (ML) bootstrap phylogenetic analysis was conducted and sequence polymorphism among individuals and populations was analyzed by calculating nucleotide diversity, the average number of nucleotide differences per site between two sequences (π; [28]). Nucleotide diversity of ITS sequences from the 12 populations ranged from 0.002 in the NY-1 population to 0.01 in the OH-1 population (Figure 2A). ITS sequence diversity within individual nematodes was also analyzed. Only a single individual nematode, WA-1.3, showed no nucleotide variation among the ITS sequences cloned (Figure 2B). Other individual nematodes showed π values ranging from 0.0006 (AR-1.1) to 0.017 (WA-1.1).

**Figure 3:**
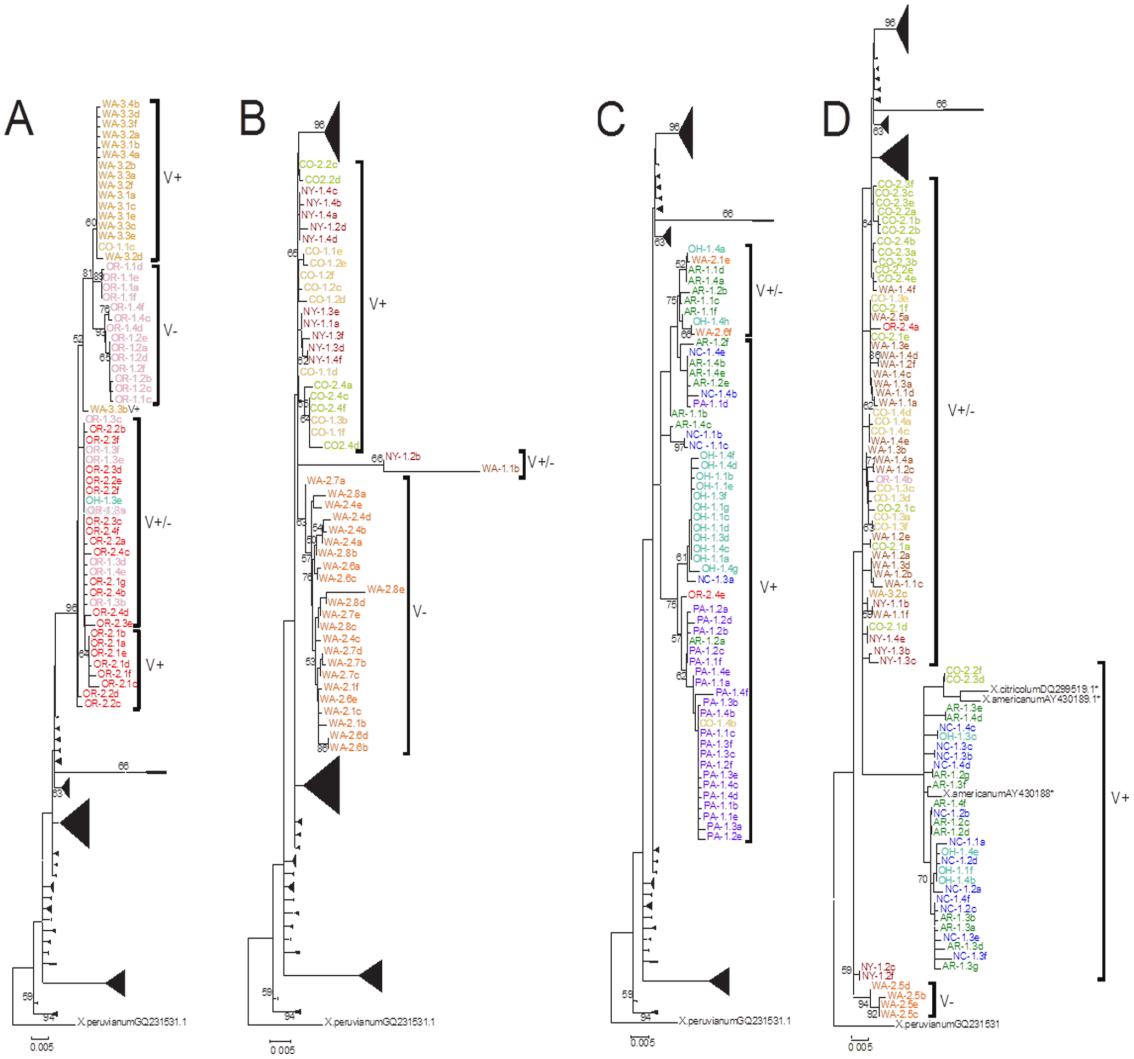
Maximum likelihood bootstrap tree of aligned ITS-rDNA gene sequences for populations of *Xiphinema americanum*, presented in 4 parts (A–D). Trees generated from the analysis of 204 newly generated ITS sequences from *X. americanum* and 4 sequences previously available from GenBank (2 of *X. americanum*, 1 *X. citricolum* and 1 *X. peruvianum*). *X. peruvianum* GQ231531.1 was designated as an outgroup. Sequence names consist of a population designation (e.g. WA-1; see Table 1) followed by a number to indicate the individual and a letter to indicate the clone (e.g. WA-1.1b is clone “b” from individual 1 from population WA-1). From 2 to 6 clones were sequenced for each individual nematode from 12 populations. Bootstrap values for clades supported at greater than 50% are shown. Brackets and V+/− indicate the presence or absence of nematode transmitted plant virus in those populations, excluding GenBank taxa indicated with an * for which virus status is unknown.

ML bootstrap analysis yielded several well-supported large clades but the relationships among most clades were poorly resolved, especially at the base of the tree (Figure 3). A clade that included most of the sequences from OR-1, OR-2 and WA-3, as well as a sequence from CO-1 and OH-1, was strongly supported (96% bootstrap value; Fig. 3A). Other sequences from these populations were found in different clades (Figure 2C, 3D). Most sequences from WA-2 formed a clade with 63% bootstrap support (Figure 3B) but other WA-2 sequences were part of different clades, including a well-supported clade at the base of the phylogeny (Figure 3D). While the bulk of OH-1 sequences were part of a moderately supported (75%) clade with all but a single sequence from PA-1 (Figure 3C), sequences from OH-1 were scattered throughout other parts of the tree (Figure 3A, 3D). Not only did sequences from the same population often fail to form a clade, sequences from individual nematodes were sometimes placed in different clades, such as sequences from individual 4 of the OR-2 population (OR-2.4a-f; Figure 3A, 3C, 3D). While some clades showed a consistent pattern of virus transmission (e.g. Figure 3A), other moderately well supported clades showed both presence and absence of virus-transmitting populations (e.g. Figure 3C). Poorly resolved relationships also resulted in a lack of clarity in virus presence or absence, especially near the base of the tree (Figure 3D).

### HTS and Phylogenetic Analyses of Mitochondrial DNA (mtDNA)

We targeted 48 individual nematodes for HTS analysis of mtDNA sequences. Using the published complete mitochondrial genome sequence for *X. americanum* as a reference [31], two sets of PCR primers were designed, each amplifying long multi-gene stretches of mtDNA (Figure 1). Together, the two amplicons amplifed nearly all *X. americanum* mtDNA sequences, with a single exception of ineffective assessment of sequence space due to primer overlap. mtDNA amplifications were successful in all 96 cases (48 nematodes × 2 amplicons/nematode), although in some instances low-abundance amplicons (weak gel bands) were produced.

After the two amplicons were generated from each of the samples, DNA was prepared for Illumina DNA sequencing using a multiplexing approach [32]. Although complete mitochondrial genome sequences resulted for many of our samples, there was extensive variation in mtDNA coverage among the 48 samples (Table S2). As a consequence, in downstream analyses we were forced to either optimize our input data set for maximum number of nematode samples included (with a trade-off of fewer mtDNA sequences included), or for maximum mtDNA positions included (with a trade-off of fewer samples).

**Figure 4:**
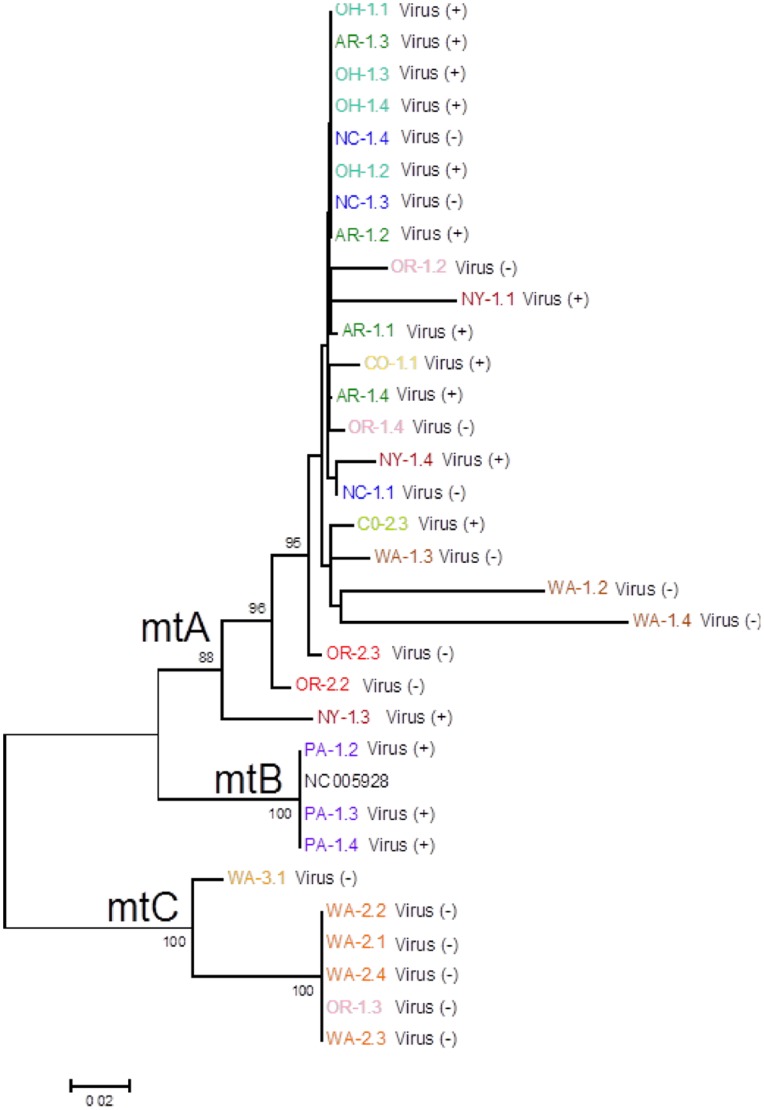
Maximum likelihood bootstrap tree of aligned ∼1.1 kb of mtDNA for populations of *Xiphinema americanum*. Thirty-two nematode sequences were analyzed. Sequence names consist of a population designation (e.g. WA-1; see Table 1) followed by a number to indicate the individual (e.g. WA-1.1). Presence or absence of nematode transmitted plant virus in an individual nematode indicated by virus (+/−).

A maximum likelihood with bootstrap analysis approach was used to phylogenetically analyze *X. americanum* mtDNA sequences resulting from the aforementioned Illumina sequencing efforts. An analysis that was optimized to include as many nematode samples as possible (32/48) that encompassed ∼1.1 kb of mtDNA and included sequence from *cytb*, *trnW*, *trnD*, and *rrnS* was performed (Figure 1). The resultant phylogeny revealed the presence of three major well-supported clades (Figure 4). The clade designated ‘mtA’ contained the largest number of nematode samples (23/32). The nematodes included in this clade were collected from diverse regions of the U.S. (9/12 total sites surveyed), including eastern (NC-1, NY-1), midwestern (AR-1, OH-1), rocky mountain (CO-1, CO-2), and pacific northwestern (OR-1, OR-2, WA-1) locations. Clade mtA contained nematodes found at sites where plant viruses vectored by *X. americanum* species complex were present (13/23), as well as samples from sites without virus (10/23). Clade mtB contained only three samples, all from a Pennsylvania site (PA-1) where virus was present. These three sequences were also identical to the GenBank entry (accession NC_005928; [31]) for the *X. americanum* complete mtDNA sequence. Clade mtC contained six samples from three different pacific-northwest regions (OR-1, WA-2, WA-3) that were all negative for virus. In some instances, we were able to analyze more than one nematode (up to 4) from a given site in this analysis. In most cases, all nematodes analyzed from a common site had identical or nearly identical sequences and were placed into a common mtDNA clade (e.g., PA-1, WA-2). However, there was one instance where the different nematodes assayed from a common site were more genetically distinct and placed into different clades: at the OR-1 site two nematode samples were placed into clade mtA and a third was placed into mtC.

**Figure 5:**
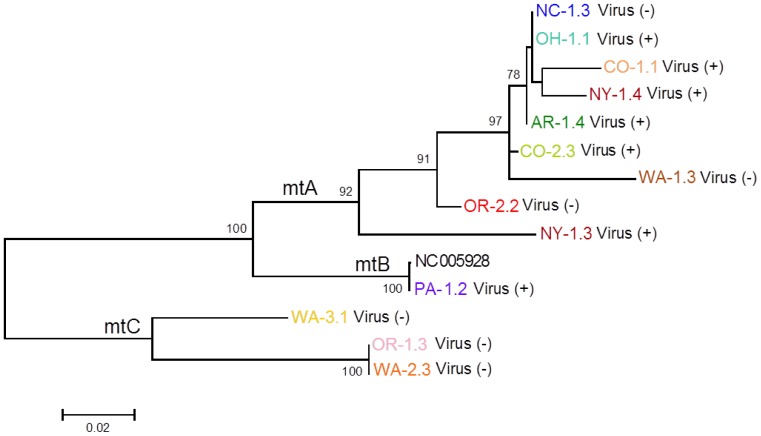
Maximum likelihood bootstrap tree of aligned ∼2.5 kb of mtDNA for populations of *Xiphinema americanum*. Thirteen nematode sequences were analyzed. Sequence names consist of a population designation (e.g. WA-1; see Table 1) followed by a number to indicate the individual (e.g. WA-1.1). Presence or absence of nematode transmitted plant virus in an individual nematode indicated by virus (+/−).

Although our analysis that optimized for a maximum number of samples provided a robust overall phylogeny with strong support of major internal branches (mtA = 88%, mtB = 100%, mtC = 100%), we performed another analysis that evaluated more mtDNA positions (∼2.5 kb) but included fewer nematode samples (n = 13 plus GenBank entry), selected based on the earlier results (Figure 5) to include representatives from the major clades and lineages in the phylogeny. The additional sequence was derived from many additional regions around the mitochondrial genome (Figure 1). The results of this second phylogenetic analysis (Figure 5) revealed a tree topology that was entirely congruent with the earlier analysis based on more nematode samples but fewer mtDNA sites. The bootstrap support remained strong in this analysis, with minor improvements at some key nodes (e.g., clade mtA was supported in 92% of bootstrap replicates here versus 88% in the analysis shown in Figure 4).

## Discussion

This study strived to bring additional clarity to the *X. americanum* species complex taxonomic conundrum by analyzing viruliferous and aviruliferous populations from a diversity of perennial cropping systems by evaluating the relative utilities of three DNA sequence types (18S rDNA, ITS, mtDNA) in illuminating relationships in this group. 18S rDNA was found to be nearly invariant among the nematodes studied, indicating that this locus evolves too slowly to be useful for clarifying relationships in the species complex. ITS1 sequences were highly polymorphic; however, this polymorphism was distributed both between populations and within individual nematodes. This pattern of polymorphism, suggesting that the ITS1 sequences do not undergo concerted evolution in these nematodes, was a significant confounder to our analysis and likely contributed to the observed limitations of this region in broadly resolving relationships among the nematodes analyzed. Although the HTS analysis was only able to recover intact mitochondrial genome sequences for a subset of the samples targeted, sufficient stretches of mtDNA shared among numerous samples were recovered and yielded robust and novel insights into *X. americanum* species complex relationships.

Despite the insights provided by our study, taxonomic clarity within *X. americanum* species complex still eludes scientists. The ramification of our inability to understand species boundaries within this group is not only an academic exercise, but has real world implications when the virus-vectoring ability of this group of nematodes is considered. It is known that members of *X. americanum* species complex from North America differentially transmit nepoviruses [8], [39] and our inability to predict when a *X. americanum* population is viruliferous or not may lead to a farmer incurring the expense of applying costly nematicides. While many nematicides have been shown to control nematodes that transmit viruses [40] the availability of, and restrictions upon many nematicides in the future will reduce their use [41]. Management practices other than the use of nematicides will be relied upon heavily in the future. Alternative control measures based on exclusion, genetic resistance, biological control, and cultural practices require a greater knowledge of nematode and virus biology to achieve satisfactory results.

18S rDNA sequence data did not provide taxonomic clarity among the populations of *X. americanum* considered in this study; only one polymorphic site was observed among the 48 nematode samples analyzed. 18S rDNA is among the slowest evolving regions within the ribosomal DNA. While this region was useful in distinguishing *X. americanum* species complex populations from other *Xiphinema* spp., it was not useful in distinguishing *X. americanum* and *X. rivesi* (a member of the *X. americanum* species complex) [38]. High 18S rDNA sequence homology among four [18], and 20 [17] *X. americanum* species complex populations further supports the limited utility in using 18S rDNA sequence data to distinguish members of *X. americanum* species complex.

Our results suggest that the ITS locus is somewhat informative for understanding the relationships between *X. americanum* species complex nematodes, but that sequence variation may be confoundingly high. We found a strongly-supported clade of sequences primarily from the WA-3, OR-1, and OR-2 populations. However, this clade also included a few sequences representing nematodes from CO-1 and OH-1 populations which had high nucleotide diversity; this may have been due to the presence of mixed populations of *X. americanum* species complex nematodes at these sites. High intra-specific and intra-individual sequence variation in the ITS region has been shown in nematodes previously [42]–[44] but few studies have broadly explored this diversity through extensive cloning. Nucleotide diversity (π) has been used to compare mtDNA pseudogene and protein-coding sequences from nematodes in *Caenorhabditis* [45] and ITS sequences from isolates of the pinewood nematode, *Bursaphelenchus xylophilus* [46]. Our high values of π were more comparable to those found for *Caenorhabditis* mtDNA protein coding genes than those of ITS sequences in *B. xylophilus*. Our sequencing of multiple clones per individual nematode allowed us to determine that the high nucleotide diversity seen in *X. americanum* species complex nematodes is primarily due to intra-individual sequence variation. These results suggest that detailed examination of intra-individual nematode ITS sequence diversity through cloning may be required before these sequences are used to address questions of phylogeny or identification.

Analysis of mtDNA sequences revealed the presence of three well-supported, mito-genetically distinct clades among the *X. americanum* species complex nematodes analyzed. These three major clades were well-supported in two different analyses of mtDNA sequence data. It is noteworthy that our analyses based on ∼1.1 kb and ∼2.5 kb of mtDNA provided highly similar and compatible results. This result suggests that the regions used in the ∼1.1 kb analysis, mostly composed of *cytb* and *rrnS* gene sequences, might provide sufficient analytical strength for future studies that incorporate larger numbers of *X. americanum* species complex populations. The COI gene sequence has received attention as an informative region of the mtDNA for *X. americanum* species complex identification [16]. It is difficult, however, to draw parallels between these two studies as the nematodes we targeted did not receive specific morphological or taxonomic attention prior to molecular analyses. The central aim of this study was to evaluate the relative utilities of different molecular targets for evolutionary analysis. Insights gained here will help guide future efforts where taxonomy and molecular phylogenic insights are more integrated.

Clade mtA contained the greatest number of samples analyzed, including nematodes from all major regions of the U.S. Nematodes in Clade mtA were collected from sites where virus-vectoring activity was detected, as well as sites where no evidence of virus was found. Interestingly, individuals from OR-1 appeared in both clades mtA and mtC indicating that a mixed population of species from the *X. americanum* species complex may have been present at this location. Clade mtB exclusively contained PA-1 (virus present) nematodes that were also identical in mtDNA sequence to the *X. americanum* complete mtDNA sequence GenBank entry. Clade mtC was found to contain highly divergent nematode mtDNA sequences. Nematodes included in this clade were collected from three different Pacific Northwest sites (OR-1, WA-2, and WA-3), all of which were negative for the presence of virus. This finding suggests that nematodes in this clade might lack or have a diminished ability to vector viruses relative to those in Clades mtA and mtB. An alternative scenario, which would not support this conclusion, is that all *X. americanum* species complex nematodes in the U.S. have the ability to vector virus, it just so happens that the virus has not been introduced extensively in the Pacific Northwest of the U.S. Regardless of the scenario, the mtDNA sequences analyzed here would provide a useful genetic screening tool for differentiating between mtC-clade nematodes and those of Clades mtA and mtB in field populations. Follow-up work that specifically looks at the relationship between mtDNA genotype, morphology, and virus vectoring ability will be required to more firmly evaluate the hypothesis presented here that mtC-clade nematodes do not vector viruses.

This study provides useful information on the relative utilities of different DNA sequences in resolving relationships among *X. americanum* species complex nematodes, indicating that mtDNA provides the most useful option. Although new phylogenetic insights in this group were achieved, a more complete understanding of *X. americanum* species complex evolutionary relationships and species boundaries will require a broader study that includes representatives of other closely-related species such as *X. pachticum and X. simile* [20]. For example, mtC might reflect a different *Xiphinema* species (relative to mtA and/or mtB), but future analyses that include other named species are required to evaluate this possibility.

## Supporting Information

Table S1 **Genbank accession entries from this study.**
(XLSX)

Table S2 **HTS mtDNA assembly results.**
(DOC)
