# Supplementary material for: Using Mitogenomic and Nuclear Ribosomal Sequence Data to Investigate the Phylogeny of the Xiphinema americanum Species Complex
Source: PLoS One. 2014 Feb 27;9(2):e90035. doi: 10.1371/journal.pone.0090035 (PMC3937401; doi:10.1371/journal.pone.0090035)
Supplement: Table S2 — HTS mtDNA assembly results. (DOC) [file pone.0090035.s002.doc]

**Table S2.** HTS mtDNA assembly results.

| **Strain** | **No. Illumina reads** | **largest mtDNA contig length** | **Assembly method** |
| --- | --- | --- | --- |
| PA-1.1 | 97253 | N/A | N/A |
| PA-1.2 | 136156 | 11749 | CLC |
| PA-1.3 | 420765 | 11438 | SCRAPE |
| PA-1.4 | 309237 | 11442 | CLC |
| WA-1.1 | 298858 | N/A | N/A |
| WA-1.2 | 141006 | 8027 | CLC |
| WA-1.3 | 275320 | 11171 | CLC |
| WA-1.4 | 191459 | 7597 | CLC |
| WA-2.1 | 284519 | 7375 | CLC |
| WA-2.2 | 65529 | 7388 | CLC |
| WA-2.3 | 327009 | 11603 | SCRAPE |
| WA-2.4 | 253033 | 7374 | CLC |
| OR-1.1 | 164659 | N/A | N/A |
| OR-1.2 | 300360 | 10435 | CLC |
| OR-1.3 | 224625 | 11603 | CLC |
| OR-1.4 | 247614 | 11132 | SCRAPE |
| WA-3.1 | 202564 | 6725 | CLC |
| WA-3.2 | 99665 | N/A | N/A |
| WA-3.3 | 83691 | N/A | N/A |
| WA-3.4 | 314665 | N/A | N/A |
| OR-2.1 | 193609 | N/A | N/A |
| OR-2.2 | 114523 | 10620 | CLC |
| OR-2.3 | 199716 | 10460 | CLC |
| OR-2.4 | 358424 | N/A | N/A |
| AR-1.1 | 488410 | 12376 | CLC |
| AR-1.2 | 473398 | 12375 | CLC |
| AR-1.3 | 434221 | 12375 | CLC |
| AR-1.4 | 486497 | 12379 | CLC |
| NC-1.1 | 573415 | 11891 | CLC |
| NC-1.2 | 451366 | N/A | N/A |
| NC-1.3 | 335175 | 12376 | CLC |
| NC-1.4 | 387116 | 11284 | CLC |
| OH-1.1 | 376179 | 12386 | CLC |
| OH-1.2 | 355306 | 11679 | CLC |
| OH-1.3 | 184628 | 12376 | CLC |
| OH-1.4 | 296699 | 12375 | CLC |
| NY-1.1 | 255195 | 10123 | CLC |
| NY-1.2 | 43802 | N/A | N/A |
| NY-1.3 | 315196 | 10890 | CLC |
| NY-1.4 | 188125 | 11284 | CLC |
| CO-1.1 | 230562 | 10850 | CLC |
| CO-1.2 | 420913 | N/A | N/A |
| CO-1.3 | 206880 | N/A | N/A |
| CO-1.4 | 306480 | N/A | N/A |
| CO-2.1 | 183922 | N/A | N/A |
| CO-2.2 | 314778 | N/A | N/A |
| CO-2.3 | 323396 | 11518 | CLC |
| CO-2.4 | 238853 | N/A | N/A |
